# Isolated cardiac metastasis from plasmacytoid urothelial carcinoma of the bladder

**DOI:** 10.1186/2162-3619-1-16

**Published:** 2012-06-24

**Authors:** Joshua R Peck, Charles L Hitchcock, Sara Maguire, Jennifer Dickerson, Charles Bush

**Affiliations:** 1 Ohio State University Wexner Medical Center, Department of Internal Medicine/Pediatrics, Columbus, OH, USA; 2 The Ohio State University Wexner Medical Center, Department of Pathology, Columbus, OH, USA; 3 The Ohio State University College of Medicine, Columbus, OH, USA; 4 The Ohio State University Richard M. Ross Heart Hospital, Department of Cardiology, Columbus, OH, USA; 5 The Ohio State University Wexner Medical Center, 452 W. 10th Ave, Room H1112, Columbus, 4321, OH, USA

**Keywords:** Plasmacytoid urothelial carcinoma, PUC, Bladder cancer, Urothelial, Isolated cardiac metastases

## Abstract

**Abstract:**

A 57-year-old male with a history of hypertension presented with shortness of breath, intermittent substernal chest pain, subjective fevers, and a 30-pound weight loss. He was found to have a bladder mass four months prior to presentation, for which he underwent cystoscopy and surgical removal. Pathology demonstrated high-grade superficial plasmacytoid urothelial carcinoma extending into the submucosa but not the muscularis propria. Given the superficial nature of his bladder cancer, a cystectomy was deferred. He was subsequently lost to follow-up care. On arrival, physical exam was notable for tachycardia, tachypnea, and distant heart sounds. An ECG showed an incomplete right bundle branch block and sinus tachycardia. Computed tomography pulmonary angiography revealed a three-cm pericardial effusion. Transthoracic echocardiography confirmed this finding and revealed a mass in the right ventricle (RV) extending into the outflow tract and infiltrating the free wall. The RV was dilated with an estimated RV systolic pressure of 37 mmHg. Pericardiocentesis yielded nearly one liter of serosanguinous fluid with non-diagnostic cytology. Partial median sternotomy with biopsy showed pathologic findings consistent with metastatic urothelial carcinoma, plasmacytoid variant. A PET scan showed increased uptake exclusively in the heart. The oncology team discussed options with the patient including chemotherapy and palliative care. The patient decided to withhold further therapy and went home with hospice care. He died two months later.

**Discussion:**

Bladder cancer is the fourth most common cancer in men in the United States. Most patients (69%) with metastatic bladder cancer have multiple organs involved; conversely, our patient had a PET scan indicating his disease was localized to the heart. Plasmacytoid urothelial carcinoma is a rare subtype of bladder cancer, and is estimated to make up less than three percent of all invasive bladder carcinomas. At the time of this publication we are aware of only three other reported instances of isolated cardiac metastasis with urothelial bladder origin; none of which were the plasmacytoid variant.

**Conclusion:**

This case highlights a previously unreported presentation of plasmacytoid urothelial carcinoma. Clinicians must remember that even superficial cancers can have significant metastatic potential.

## Introduction

Urothelial cell carcinoma (UCC) with metastasis exclusively to the heart is an extremely rare and atypical presentation of a relatively common malignancy. A review of current literature yields only three other instances of this unusual metastatic pattern [1-3]. We present a 57-year-old male with plasmacytoid urothelial carcinoma (PUC) of the bladder and isolated metastasis to the heart. To our knowledge, an isolated cardiac metastasis from this rare type of UCC has never been reported in the literature.

### Case report

A 57-year-old male with a past medical history of hypertension, type-2 diabetes and superficial UCC presented with shortness of breath, substernal chest pain, and fevers. He was found to have a bladder mass four months earlier, for which he underwent cystoscopy and surgical removal. Pathology demonstrated high-grade superficial PUC extending into the submucosa but not the muscularis propria. Given the superficial nature of his bladder cancer, a cystectomy was deferred. He was subsequently lost to follow-up care.

**Figure 1:**
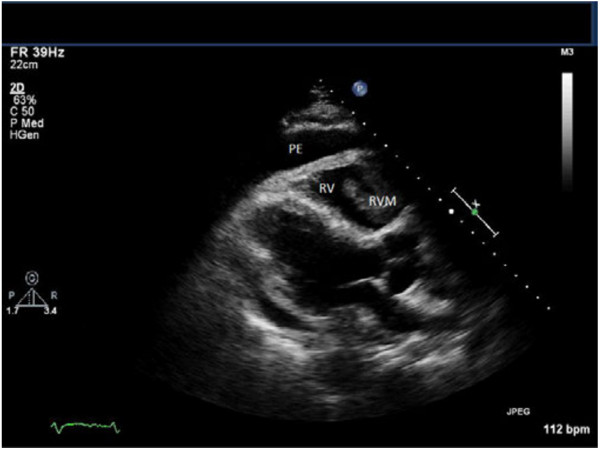
Transthoracic Echocardiogram in a Parasternal Long Axis View showing a Large Pericardial effusion and RV Mass Large Pericardial effusion and RV Mass.

**Figure 2:**
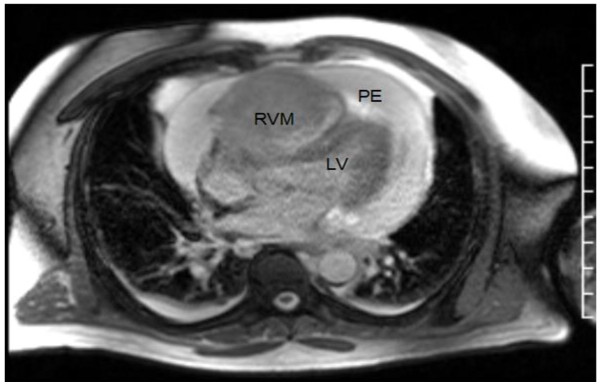
Cardiac MRI, T1 weighted bright blood imaging axial orientation.

**Figure 3:**
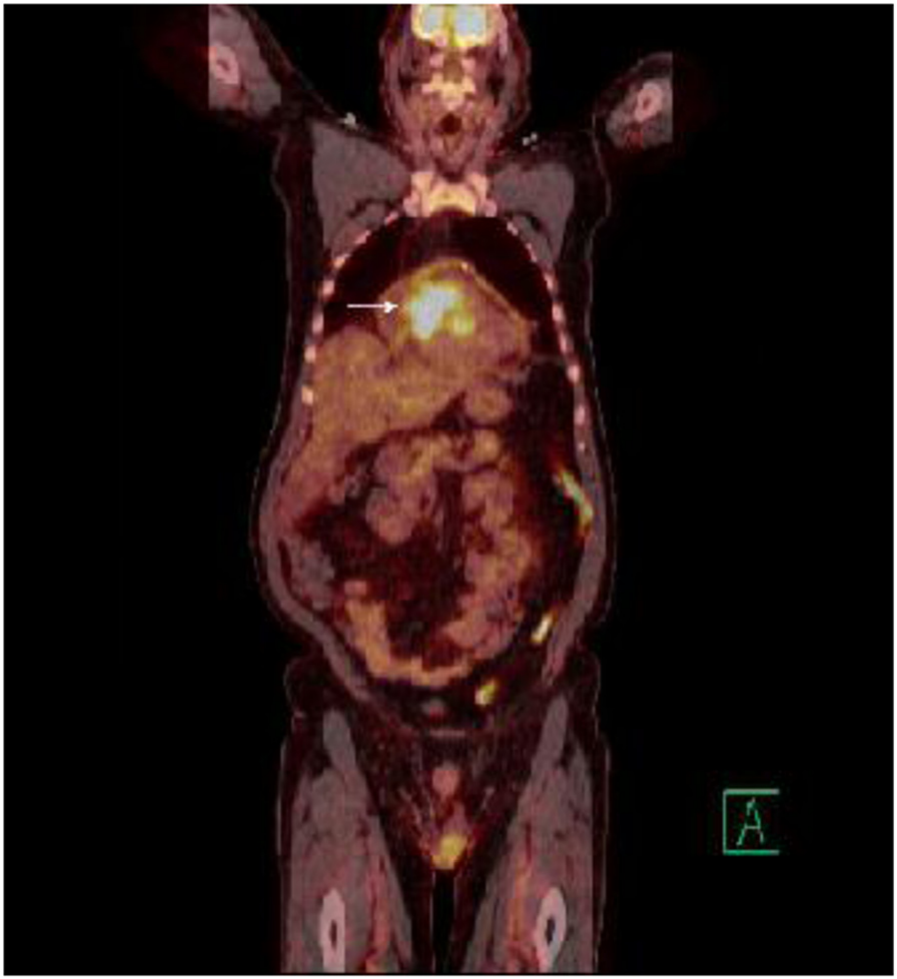
PET scan showing intense uptake in the myocardium (arrow).

Upon presentation to our emergency department, the patient was tachycardic and tachypneic with distant heart sounds. An electrocardiogram (ECG) showed an incomplete right bundle branch block and sinus tachycardia. A computed tomography (CT) pulmonary angiography scan revealed a three-cm pericardial effusion. Transthoracic echocardiography visualized a right ventricular (RV) mass which extended into the outflow tract and appeared to infiltrate the RV free wall (Figure 1). A large pericardial effusion was present. The RV was dilated with an estimated RVSP of 37 mm Hg. There was no sign of right heart collapse. Subsequent T1 weighted cardiac Magnetic Resonance Imaging (MRI) in the axial view showed the RV free wall diffusely infiltrated with tumor extending into the RV cavity and a large circumferential pericardial effusion (Figure 2). Pericardiocentesis yielded nearly one liter of serosanguinous fluid with non-diagnostic cytology. A whole body Positron Emission Tomography (PET) scan showed increased uptake exclusively in the heart (Figure 3).

**Figure 4:**
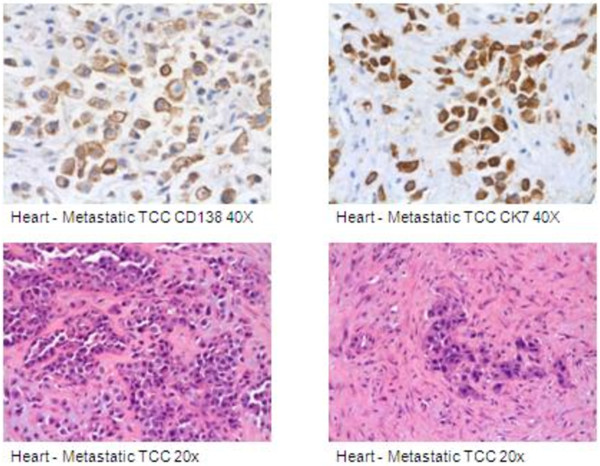
Right Ventricular Mass Biopsy.

A mini sternotomy with biopsy showed infiltrating tumor cells with a plasmacytoid appearance and a strong desmoplastic response (Figure 4). Immunohistochemical staining demonstrated tumor cells consistent with metastatic PUC.

The oncology team discussed options including chemotherapy, palliative surgical debulking, and comfort care. The patient decided to go home with hospice. He died two months later.

## Discussion

Our discussion highlights the uniqueness of bladder cancer metastasizing to the heart and explores the rare subtype of PUC. Bladder cancer is the fourth most common cancer in men in the United States [4]. Normal patterns of metastasis typically follow a predictive pattern, first involving regional and juxtaregional lymph nodes, then liver, lungs, bones, and less commonly intestine, adrenal glands and kidneys [5]. Most patients (69%) with metastatic bladder cancer have more than one organ involved [6]. Cardiac metastases are much less common, with the first case reported in 1967 [7]. At the time of this publication we are aware of only three other reported instances of isolated cardiac metastasis from urothelial cell carcinoma [1-3].

Metastases to the heart and pericardium are incidentally discovered at autopsy in 10%–12% of patients with any type of malignancy [8]. Primary tumors most likely to have cardiac metastasis found at autopsy include pleural mesotheliomas (48.4%), melanomas (27.8%), lung adenocarcinomas (21%), and undifferentiated carcinomas (19.5%) [9]. It is very rare for malignancies to have solitary cardiac metastasis, with post-mortem studies finding rates of only 0.015%.

PUC is a rare and only recently described type of bladder cancer, with the first reported case in 1991 [10]. It is estimated to make up less than three percent of all invasive bladder carcinomas [11]. Although it is still a relatively new entity, data thus far has demonstrated that PUC is an aggressive tumor with extensive local growth and poor prognosis [12].

The histopathology of PUC is characterized by invasive discohesive growth of plasmacytoid cells with eccentric nuclei. The normal immunohistochemical staining pattern for urothelial carcinomas is variable. Cytokeratin (CK) 7 staining occurs in the majority of these tumors; whereas as the CK20 expression varies from 15% to 97% depending on the study [13]. PUC cells stain with CK, epithelial membrane antigen, GATA binding protein 3 (endothelial transcription factor 3), cluster of differentiation (CD) 15, CD138, protein 53 (p53), and protein 16 (p16) [14-16].

**Figure 5:**
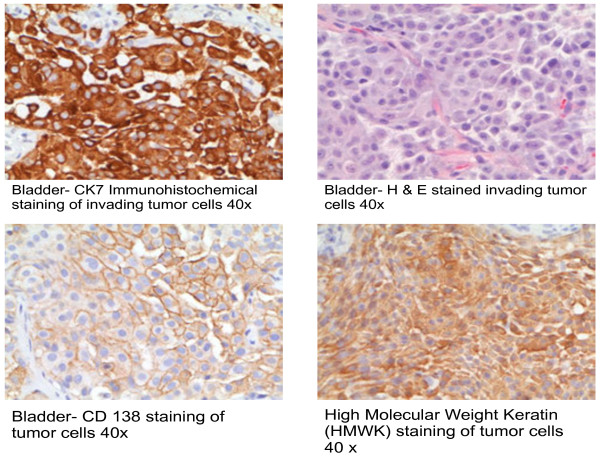
Bladder Mass Biopsy.

A review of hematoxylin and eosin (H&E) stained sections of the biopsied primary tumor demonstrated an invasive urothelial carcinoma with discohesive epithelial cells and plasmacytoid features (Figure 5). Subsequent immunohistochemical staining demonstrated positive cytoplasmic staining for pankeratin anion exchanger 1 and 3 (AE1/AE3, not shown), high molecular weight keratin (HMWK), CK7, and positive cell surface staining for CD138. All of the tumor cells had this staining pattern. The same cells were seen in the cardiac biopsy (Figure 4) along with a prominent desmoplastic response not seen in the primary tumor. Immunohistochemical staining of the cardiac biopsy demonstrated positive staining for AE1/AE3, HMWK, CK7, and CD138. Although not performed on the primary tumor, the metastatic cells lacked staining for CK20, muscle-specific actin (HHF-35), myogenin, desmin, calretinin, the monoclonal antibody D2-40, Wilm’s tumor gene (WT-1), Melan-A, protein S-100, Human Melanoma Black (HMB-45), and CD45.

In general, the differential diagnosis for a CK7 positive and CK20 negative tumors include urothelial carcinoma, breast carcinoma, ovarian serous carcinoma, mesothelioma and prostatic carcinoma. In this case, the differential diagnosis is narrowed to urothelial carcinoma, mesothelioma and prostatic carcinoma. There was no clinical or radiographic evidence of mesothelioma, nor was there positive staining of the metastatic tumor for the mesothelial markers calretinin, WT-1, and D2-40. High molecular weight cytokeratin is highly sensitive marker for urothelial carcinomas and helps distinguish it from prostate carcinoma which lacks HMWK expression.

Cardiac magnetic resonance imaging was conducted to further image the myocardium to obtain information regarding the histopathologic composition of the tumor as well as to evaluate for any adverse hemodynamic effects. Cardiac MRI is superior for assessment of tumor morphology to assist with mass characterization. While hybrid PET/CT has been used for imaging of solid organs, this has not been widely used for cardiac imaging [17,18].

Metastases from PUC have been reported in adjacent pelvic structures, bone, and peritoneum. [12,19]. In a review of the literature, we have not identified any cases of isolated or non-isolated cardiac metastasis from this subtype of UCC. It is unknown why there have not been any other documented cases of metastasis to the heart, however a possible explanation may be that this tumor is usually spread via direct extension into surrounding structures rather than hematogenously.

PUC is often discovered at a late stage, which contributes to its poor prognosis. Our patient had a superficial PUC that did not invade the muscularis propria of his bladder wall. Conservative treatment was chosen for the patient based on these findings. Despite removal of any identifiable whether the initial biopsy report underestimated the staging, or whether tissue had already seeded the heart. Whole body PET suggested that the PUC did not persist in his bladder and had not metastasized to any other sites. Such findings point to the need for more aggressive interventions and close follow-up for patients with the plasmacytoid variant of UCC, regardless of the degree of tumor infiltration.

## Consent

Written informed consent was obtained for publication of this report and any accompanying images from the patient’s next-of-kin.

## Competing interests

The authors declare that they have no competing interests.

## Authors’ contributions

JP and SM performed the computerized literature search. CH stained and interpreted the Pathology slides. JD interpreted the echocardiogram. JP and CB conceived the paper. JP, SM, JD, CH, and CB all participated equally in drafting the manuscript. All authors read and approved the final manuscript.
